# *Brucella ceti* infection in dolphins from the Western Mediterranean sea

**DOI:** 10.1186/s12917-014-0206-7

**Published:** 2014-09-17

**Authors:** Marcos Isidoro-Ayza, Nazareth Ruiz-Villalobos, Lola Pérez, Caterina Guzmán-Verri, Pilar M Muñoz, Fernando Alegre, Montserrat Barberán, Carlos Chacón-Díaz, Esteban Chaves-Olarte, Rocio González-Barrientos, Edgardo Moreno, José María Blasco, Mariano Domingo

**Affiliations:** Departament de Sanitat i Anatomia Animal, Facultat de Veterinària, Universitat Autònoma de Barcelona (UAB), and Servei de Diagnòstic de Patologia Veterinària, Facultat de Veterinària, Universitat Autònoma de Barcelona (UAB), 08193 Bellaterra, Barcelona, Spain; Programa de Investigación en Enfermedades Tropicales, Escuela de Medicina Veterinaria, Universidad Nacional, Heredia, Costa Rica; CITA Gobierno de Aragón, 50059 Zaragoza, Spain; Centre Fundación para la Conservación y Recuperación de Animales Marinos (CRAM), El Prat de Llobregat, Barcelona, Spain; Centro de Investigación en Enfermedades Tropicales, Facultad de Microbiología, Universidad de Costa Rica, San José, Costa Rica; Ministerio de Agricultura y Ganadería, Laboratorio de Patología, Heredia, Costa Rica; Instituto Clodomiro Picado, Universidad de Costa Rica, San José, Costa Rica; Centre de Recerca en Sanitat Animal (CReSA), UAB-IRTA, 08193 Bellaterra, Cerdanyola del Vallès, Barcelona, Spain

**Keywords:** *Brucella ceti*, Cetacean, Neurobrucellosis, Discospondylitis, Mediterranean sea

## Abstract

**Background:**

*Brucella ceti* infections have been increasingly reported in cetaceans. Brucellosis in these animals is associated with meningoencephalitis, abortion, discospondylitis’, subcutaneous abscesses, endometritis and other pathological conditions *B. ceti* infections have been frequently described in dolphins from both, the Atlantic and Pacific Oceans. In the Mediterranean Sea, only two reports have been made: one from the Italian Tyrrhenian Sea and the other from the Adriatic Sea.

**Results:**

We describe the clinical and pathological features of three cases of *B. ceti* infections in three dolphins stranded in the Mediterranean Catalonian coast. One striped dolphin had neurobrucellosis, showing lethargy, incoordination and lateral swimming due to meningoencephalitis, A *B. ceti* infected bottlenose dolphin had discospondylitis, and another striped dolphin did not show clinical signs or lesions related to *Brucella* infection. A detailed characterization of the three *B. ceti* isolates was performed by bacteriological, molecular, protein and fatty acid analyses.

**Conclusions:**

All the *B. ceti* strains originating from Mediterranean dolphins cluster together in a distinct phylogenetic clade, close to that formed by *B. ceti* isolates from dolphins inhabiting the Atlantic Ocean. Our study confirms the severity of pathological signs in stranded dolphins and the relevance of *B. ceti* as a pathogen in the Mediterranean Sea.

**Electronic supplementary material:**

The online version of this article (doi:10.1186/s12917-014-0206-7) contains supplementary material, which is available to authorized users.

## Background

After the first descriptions of *Brucella* infections in dolphins and seals and the definition of *Brucella ceti* and *Brucella pinnipedialis* as two new species within the genus [1-3], there has been an increasing recognition of brucellosis in marine mammals (see [4] and [5] for recent reviews). *Brucella* strains from marine mammal origin have been isolated from humans [6]. Antibodies against *Brucella* have been detected in 28 out of 42 cetacean species investigated, and *B. ceti* has been isolated from 10 of these species [5]. *B. ceti* infection in cetaceans is associated to meningoencephalomyelitis [1,3,7-9], abortion [1,2], discospondylitis, subcutaneous abscesses, endometritis, and a wide range of other pathological conditions [4,5,10]. However, with the exception of the striped dolphin *(Stenella coeruleoalba)* [1,3-5,9-13] the proportion of other cetacean species showing clinicopathological signs associated with brucellosis is low, suggesting that most of these infected animals overcome clinical disease, eventually remaining as *Brucella* carriers and shedders.

Presumptive *Brucella* infections in Western Mediterranean Sea dolphins was first established by serology in two striped dolphins and one bottlenose dolphin (*Tursiops truncatus)* stranded on the Mediterranean Catalonian coast [14]. Recently, *Brucella* strains were isolated from striped dolphins in the Tyrrhenian and Adriatic Seas [11,15]. Here, we describe the clinical and pathological features of three cases of brucellosis in dolphins stranded on the Mediterranean Catalonian coast, and provide detailed information on the phenotypic and molecular characterization of these three *B. ceti* isolates.

## Methods

### Dolphin stranding and serological, pathological and bacteriological examinations

Two striped dolphins (N-372/09, N-301/12) and one bottlenose dolphin (N-275/12) reacting positive in standard Rose Bengal Test (RBT), performed according to standard procedures [16] were included in this study. The relevant animal and stranding data are shown in Table 1. At the time of necropsy all three dolphin carcasses were in a good preservation state (2 in a scale of 1 -very good- to 5 -poor-). Gross pathological studies were performed in the three dolphins and complete sets of organs and tissues for each animal were preserved in 10% buffered formalin and processed for histopathological examination and immunohistochemical detection of Cetacean Morbillivirus (CeMV) as described previously [17]. Selected samples of brain tissue and spleen from the three dolphins were also examined immunohistochemically using an anti-*B. melitensis* 16 M polyclonal rabbit antiserum and avidin-biotin peroxidase system as described elsewhere [7,12].

**Table 1:** **Biological data of** ***Brucella ceti*** **infected Mediterranean dolphins**

**Identification**	**Place, coordinates and date of stranding**	**Pathological diagnoses (Macro/Micro)**	**Tissues sampled for bacteriological examination**	***B. ceti*** **strain isolated from**
N-372/09	Salou, Spain	Non-suppurative encephalitis by CeMV. RT-PCR and IHC for CeMV both positive only in CNS	Encephalon, spleen, diaphragmatic and preescapular lymph nodes, lung	bmarMR26
*Stenella coeruleoalba*	(41.0733, 1.1343)			Spleen
Male, 1.94 m, 74.5 Kg	Found alive Sept 11th-2009			
N-275/12	Badalona, Spain	Mycotic pyogranulomatous-necrotizing meningoencephalomyelitis	Encephalon, CSF (swab from lateral ventricle), vertebral abscess (swab), spleen.	bmarMR25
*Tursiops truncatus*	(41.4458, 2.2507)			Vertebral abscess
Male 3 m	Found dead 7May 23rd-2012	Chronic, severe, focally extensive, suppurative discospondylitis		
		RT-PCR and IHC for CeMV negative		
N-301/12	Cunit, Spain	Non-suppurative meningoencephalitis. RT-PCR and IHC for CeMV negative	Encephalon, spleen, diaphragmatic lymph node, lung	bmarMR24
*Stenella coeruleoalba*	(41.1931, 1.6360)			Encephalon
Female, 1.84 m, 54.5 Kg	Found alive June 3rd-2012			

Tissue samples of two dolphins (N-275/12 and N-301/12) were collected at the time of necropsy and submitted for bacteriological examination (Table 1). Tissues of the third dolphin (*S. coeruleoalba,* N-372/09), frozen at −80°C since 2009, were defrosted and submitted also to bacteriological studies, but cerebrospinal fluid (CSF) was not available in this case. Swabs taken at necropsy were each smeared in at least two plates of both Farrell’s [18] and CITA [19] culture media. The remaining necropsy samples were homogenized under sterile conditions in the minimum amount possible of sterile buffered saline (PBS pH 6.8) in a Stomacher unit (Seward Medical, Worthing, UK), and 0.5 mL of each tissue homogenate seeded also on at least two plates of each selective culture medium. The plates were checked for growth after 5–8 days of incubation at 37°C both in air and 10% CO_2_ atmospheres. *Brucella* colonies were identified by colonial morphology and standard typing procedures [20,16]. One culture was considered as positive when at least one *Brucella* colony forming unit (CFU) was isolated. The suspected *Brucella* colonies isolated were further identified and characterized by molecular and chemical methods (see below).

### Control strains

The following strains obtained from the CITA and from PIET/CIET strain collections were used as controls for molecular studies: *B. ceti* Atlantic dolphin type (B14/94), *B. ceti* Atlantic porpoise type (B1/94), *B. ceti* Cantabric Sea isolate from *S. coeruleoalba* stranded in Northern Spain (C1), *B. pinnipedialis* seal type (B2/94), *Brucella abortus* 2308 (biovar 1 virulent reference strain), *B. abortus* S19 (biovar 1 reference vaccine strain), *Brucella melitensis* Rev1 (biovar 1 reference vaccine strain), *Brucella suis* (S2 biovar 1 ), *B. canis* (CR206-10; Costa Rica isolate), *B. neotomae* 5 K33 (reference strain), *Brucella ovis* PA (virulent reference strain) and *Brucella microti* (CCM4915, reference strain).

### Molecular studies

*Brucella* DNA samples from each isolate and control strains were extracted with DNeasy Blood & Tissue kit from QIAGEN®, and stored at −70°C until used. The three Mediterranean dolphin isolates were identified as *B. ceti* using the multiplex PCR as described elsewhere [21]. DNA samples from these isolates and the marine control strains were also tested by PCR-RFLP of *omp2b* locus [22] and by multiplex PCR using the following two pairs of primers: TCA ACT GCG TGA ACA ATG CT (f) / GCG GGC TCT ATC TCA AGG TC (r), and CGT CAA CTC GCT GGC CAA GAG (f) / GCA GGA GAA CCG CAA CCT AA (r). Multiple loci variable number of tandem repeats (MLVA-16) analysis of *Brucella* species and strains was performed as described previously [23-26]. The basic protocol for MLVA-16 was slightly modified to use DreamTaq™ PCR Master Mix (Fermentas®). Amplicon analysis was performed on the ChemiDoc Gel Documentation System XRS, BioRad® using the Quantity One® software, which allowed molecular size determination of amplicons. *Brucella* control strains were used for validation [12,23,26]. The profiles were entered in the database MLVA-NET for the corresponding analysis [27].

### Mass spectrometry analysis of *Brucella* protein extracts

For MALDI-TOF studies, the three *B. ceti* isolates and all control strains were grown in trypticase soy agar plates for four days in the presence or in the absence of CO_2_ (as required) following modifications of previous protocols [16,18,28]. For each bacterial strain, three clearly separated colonies were suspended in a 1.5 ml Eppendorf tube containing 1 ml of ultrapure distilled water, and centrifuged at 14,000 rpm for 5 min. Then, the bacterial pellet was thoroughly resuspended in 300 μL of water. After this, 700 μL of absolute ethanol were added, the suspension mixed in a vortex, let rest for five minutes at room temperature and centrifuged at 14,000 rpm for 5 minutes. The bacterial pellet was resuspended in 250 μL of water and directly sonicated in the Eppendorf tube with the aid of a titanium micro tip, at room temperature for 2 minutes. Nine hundred μL of absolute ethanol were added and the extract dried to completeness in a speed-vac centrifuge at 45°C for ~2.5 hours. Fifty μL of 70% formic acid were added to suspend the dried pellet, mixed thoroughly by pipetting and then 50 μL of acetonitrile were added and mixed. The extract was centrifuged at 14,000 rpm for 5 minutes and the supernatant transferred into a clean tube. In order to select optimal conditions for mass spectrometer analysis, several dilutions of the extract were tested. A volume of 0.5 μL of each dilution was spotted onto a steel Opti-TOF 384 plate target (ABSciex) and air-dried at room temperature. The spot sample was overlaid with 0.5 μL of matrix solution (saturated solution of alpha-cyano-4-hydroxy-cinnamic acid) in organic solvent (50% acetonitrile and 2.5% trifluoroacetic acid) and air-dried. The samples were analyzed in a MALDI-TOF on an Applied Biosystems 4800 Plus mass spectrometer. Spectra were acquired in linear positive mode, using a laser intensity of 3,800 and 500 shots/spectrum, in the m/z range 2,000 to 11,000, after external MS calibration with CalMix-5 standards (ABSciex) spotted on the same plate. Spectra were visualized using Data Explorer v.4.9 (Applied Biosystems).

### Gas chromatographic analysis of fatty acid methyl esters

The three *B. ceti* isolates and all control strains were grown as described above. For each bacterial strain, 75 clearly separated colonies were chosen from three plates and placed in a sealed glass tube. Saponification of the samples and processing for total fatty acid methyl ester determination were carried out according to the MIDI instruction manual of Technical Note #101 (MIS, MIDI Inc., Newark, DE). Analysis was performed by gas chromatography (Agilent Technologies 6850) using a 25 m x 0,2 mm cross linked phenyl-methyl silicone fused silica capillary column HP 19091B-102 (Agilent Technologies Inc., Santa Clara, CA). A binary matrix was generated using the fatty acid profile of the tested strains.

### Phylogenetic analysis and cladograms

Dendrograms based on the retention time of the fatty acid methyl esters and on the protein masses detected were constructed using an Agglomerative hierarchical clustering (AHC) algorithm, using Microsoft® Excel 2000/XLSTAT^©^-Pro (Version 4.07*,* 2013, Addinsoft, Inc., Brooklyn, NY, USA). Proximities were calculated using Squared Euclidean Distance, and aggregation was calculated using the unweighted pair-group average method. MLVA 16 phylogenetic trees based on differences in MLVA-16 was built according to the procedures described in the *Brucella* MLVA database [27].

## Results

### Clinical and pathological findings

The main biopathological features of the three Mediterranean dolphins from which *B. ceti* strains were isolated are summarized in Table 1.

**Figure 1:**
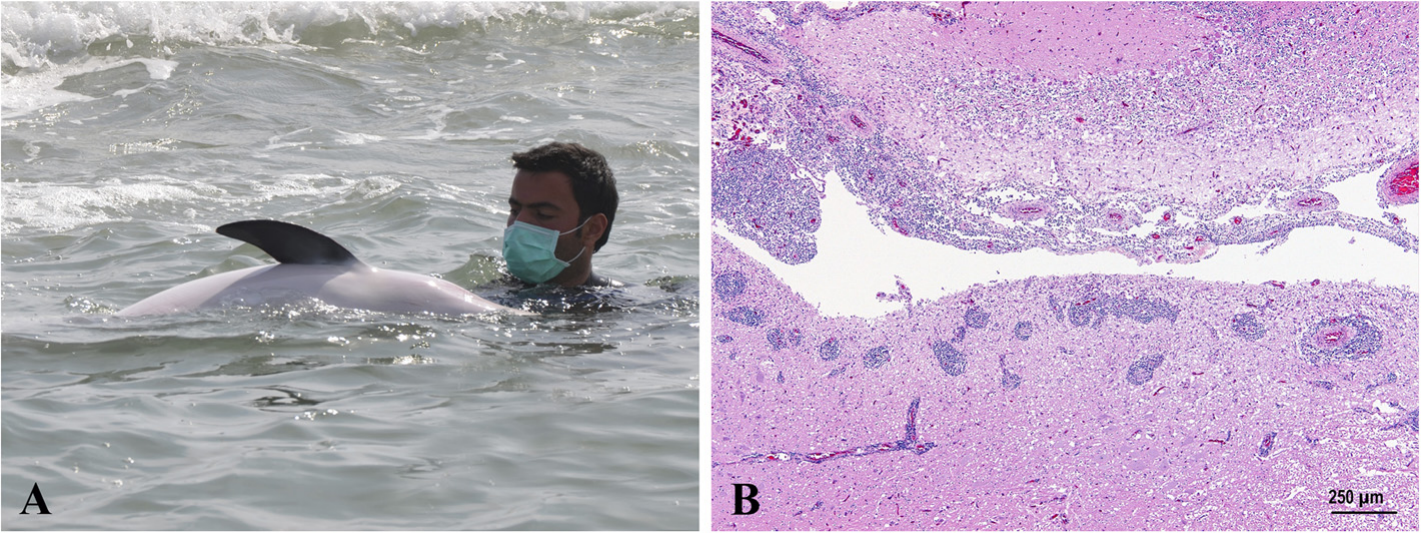
**Striped dolphin, case N-301/12. A**. Dolphin with clinical signs of neurobrucellosis, showing lateralization when swimming and inability to maintain equilibrium and flotation. The dolphin needed continuous holding and support. **B**. Histopathologic section of the brain from the same case. Thick perivascular infiltrates mainly composed of lymphocytes, plasma cells and macrophages are seen affecting leptomeninges, the neuropil surrounding the fourth ventricle and the choroid plexus (H&E).

Striped dolphin N-301/12 was found stranded alive, showing uncoordinated swimming, with circling and severe lateralization, needing continuous support (Figure 1A). This dolphin died four days after stranding, in spite of the supportive medical care. Episodes of tonic-clonic seizures were observed shortly before death. At necropsy, this animal showed a good body condition. No significant gross lesions were observed, apart from a moderate amount of subcutaneous oedema in the cranial region of the trunk, and multiple erosions of the tongue epithelium. A moderate infestation of the caudal blubber by larval forms of the tapeworm *Phyllobothrium delphini* and bile and pancreatic ducts by trematodes of the genus *Campula* were observed. These are common parasites of striped dolphins. Histologically, the most relevant finding was a severe, diffuse, chronic, non-suppurative, meningoencephalomyelitis. The inflammatory process affected the leptomeninges, and the subependymal neuropil (periventriculitis), forming thick perivascular cuffs that shallowly penetrated into the underlying grey and white matter (Figure 1B). The process was more severe in cerebellum, brainstem, spinal cord and medulla oblongata and with less involvement of the cerebral cortex. The inflammatory infiltrate was composed of a large number of lymphocytes and plasma cells and a lesser number of macrophages. A similar inflammatory infiltrate was observed in the choroid plexus (choroiditis) as well as in the dorsal and ventral cervical nerve roots (radiculitis). All studied sections of the nervous system showed variable degrees of gliosis, satellitosis with formation of glial nodules, spongiosis and perivascular oedema.

Small, multifocal, randomly distributed, non-suppurative inflammatory infiltrates were found in the liver and kidney. In addition, a small focus of granulomatous and necrotizing lymphadenitis was seen in the mesenteric lymph node. These lesions were probably associated to parasitic migrations. No other relevant microscopical findings were found in examined tissues. *Brucella* antigens were not detected by IHC in CNS. RT-PCR for CeMV resulted negative in all the investigated tissues.

**Figure 2:**
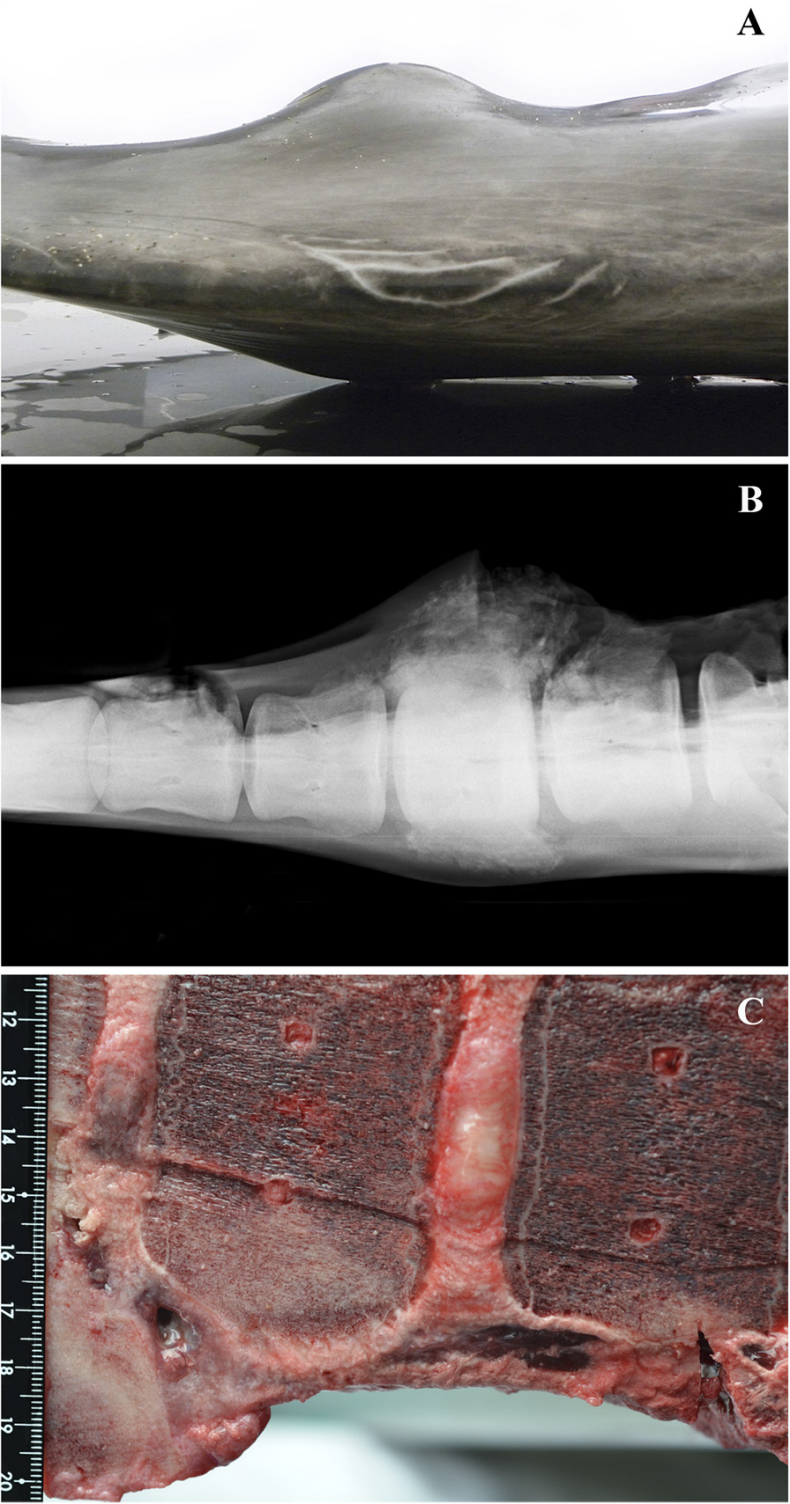
**Bottlenose dolphin, case N-275/12. A**. Mass in the caudal peduncle, dorso-ventral view of the lesion at necropsy. **B**. Radiograph from the lesion in dorso-ventral view, showing periostal proliferation with ankylosis of coccygeal vertebrae. **C**. Longitudinal section of the affected vertebra, showing bony proliferation at the ventrolateral side of the vertebral body, leading to ankylosis. There is formation of small abscesses. *Brucella ceti* was cultured from one of the abscesses.

The bottlenose dolphin (N-275/12) was found dead. Gross external examination revealed that this dolphin was in a good body condition. Several “Tattoo’-like lesions measuring 2–4 cm in diameter were observed on the skin of the left maxilla. A hard spherical mass of approximately 18 cm in diameter was found in the caudal peduncle. The skin was unaffected at this site (Figure 2A). On radiographs the mass was characterized as a marked bone proliferation rising from the periosteum of two consecutive coccygeal vertebrae generating ankylosis of the implicated joint (Figure 2B). A longitudinal section (Figure 2C) showed a chronic, severe, focally extensive and suppurative disco-spondylitis with marked disruption of the adjacent soft tissues (skeletal muscle fascicles, tendons and fascia). Histopathology from this lesion was not performed. In the CNS, a slightly increased amount of CSF was noticed at the lateral ventricles. After pre-fixation, the brain was sliced. At that time, poorly demarcated, bilateral areas of malacia where identified in the area of the *nucleus caudatus*. A third focus of malacia was disclosed in the area of the *nucleus accumbens* of the left hemisphere. Microscopically, there was a pyogranulomatous and necrotizing meningoencephalomyelitis and radiculitis caused by the fungus *Cunninghamella bertholletiae* that has been described in detail elsewhere [29]. No other significant lesions were found at necropsy in this dolphin. Tissues tested by RT-PCR for CeMV yielded negative results. IHC for *Brucella* yielded negative results at CNS. The coccygeal diskospondylitic lesion was not investigated by IHC.

**Figure 3:**
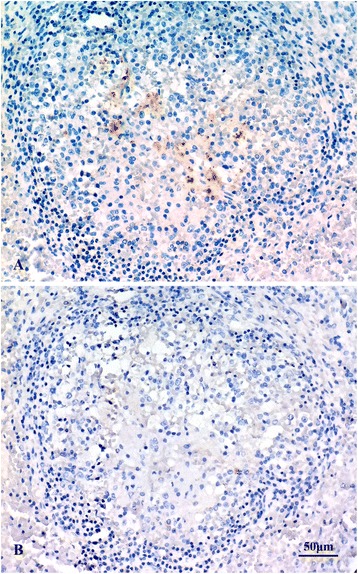
**Immunohistochemical staining of** ***Brucella*** **, striped dolphin (case 372/09), spleen section. A**. Section incubated with specific polyclonal antiserum to *Brucella melitensis*. Positivity was found in cytoplasm of cells within lymphoid follicles. **B**. Parallel section of the same tissue incubated with control negative antiserum. Haematoxylin counterstain.

Striped dolphin N-372/09 was found alive showing lethargy, incoordination and lateral swimming. The animal died before the rescue protocol could be initiated. At necropsy, no significant macroscopic lesions were observed in this dolphin, except for the absence of content in the stomach cavities. Moderate infestation by the common larval forms of the tapeworms *Phyllobothrium delphini* and *Monorygma grimaldi* were also observed in the caudal blubber and the peritoneal cavity respectively. Histopathological examination revealed chronic, non-suppurative encephalitis as the main pathological sign. Immunohistochemical (IHC) staining for CeMV detected viral antigen at the Central Nervous System (CNS) of this dolphin. This result was confirmed by a CeMV positive RT-PCR in CNS tissue. Consequently, this case was diagnosed as a CNS-localized chronic CeMV infection [30]. IHC for *Brucella* antigens showed positive staining in cytoplasm of cells inside splenic lymphoid follicles (Figure 3).

### Bacteriological findings

Bacterial isolates identified as *Brucella* strains were obtained in high numbers from the spleen (N-372/09), vertebral abscess (N-275/12) and brain (N-301/12), respectively. The strains were isolated in the presence and absence of CO_2_ and selective culture media with the exception of strain from case N-301/12, whose growth was inhibited on Farrell’s medium. The isolated strains were not capnophilic, were positive for the oxidase and urease tests, and displayed a smooth type, agglutinating with both anti-A and anti-M mono-specific sera. The strains were as well lysed by the Iz but not the Tb, Wb and R/C phages, and grew on standard concentrations of both thionin and basic fuchsin. The three Mediterranean isolates were identified as *B. ceti* using the multiplex PCR. They were named as bmarMR24 (isolated from case N-301/12), bmarMR25 (from case N-275/12) and bmarMR26 (from case 372/09).

### MLVA16, protein and fatty acid polymorphisms and phylogeny

**Figure 4:**
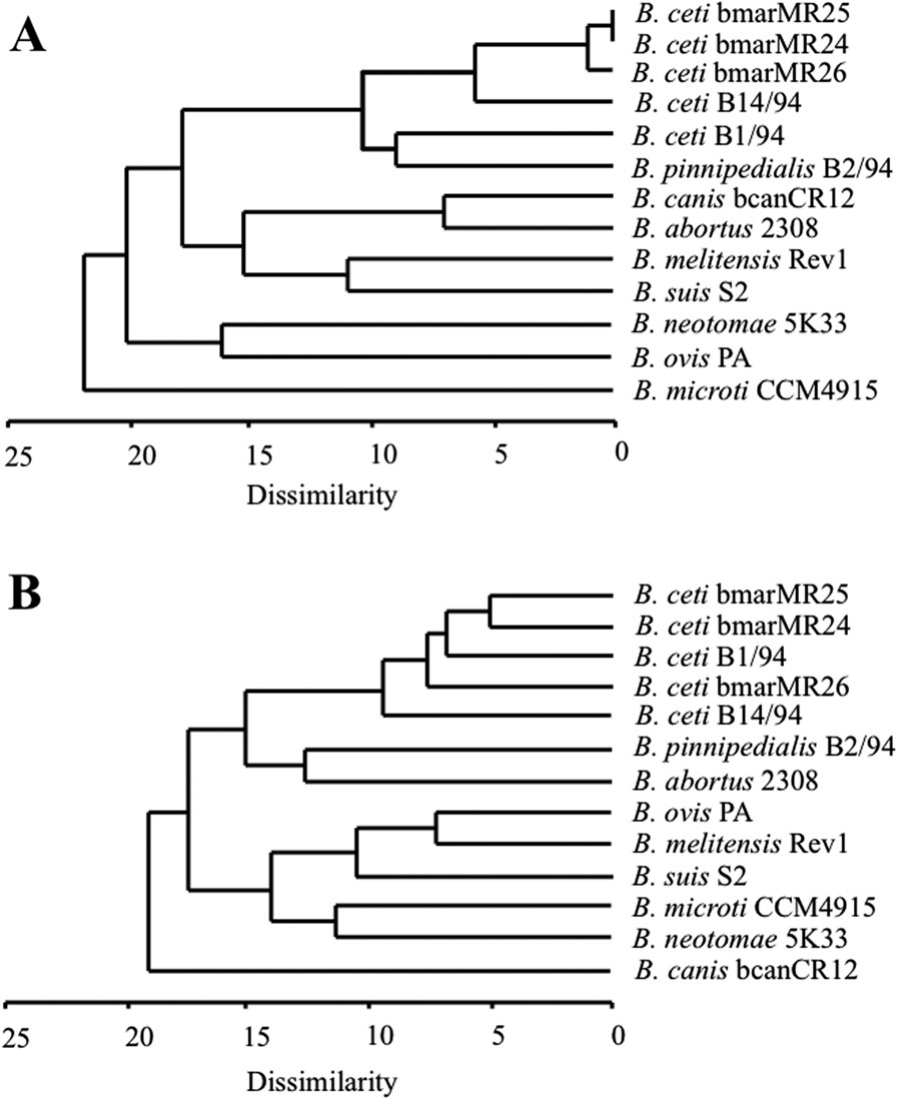
**Dendrograms based on MALDI-TOF analysis of protein molecular weight (A) and gas liquid chromatography analysis of the fatty acid methyl esters (B) of different** ***Brucella*** **extracts.** Notice that in “A” the three Mediterranean strains cluster together in a single group; while in “B” they are intertwined with a *B. ceti* isolate from a striped dolphin from the Atlantic Ocean.

Fatty acid and protein molecular phenotyping are well recognized methods for typing bacteria and have been used extensively to differentiate bacteria, including *Brucella* strains [24,31,32]. The MLVA16 respective sizes for the analysed strains, the protein molecular weight peaks determined by MALDI-TOF and the retention times of the fatty acid methyl ester peaks determined by GLC of the strains studied are presented respectively in Additional file 1: Tables S1, Additional file 2: Table S2, and Additional file 3: Table S3. Dendrograms built on the basis of MALDI-TOF analysis demonstrated that the three *B. ceti* Mediterranean isolates clustered together in a distinct clade, close to the Atlantic dolphin type B14/94 strain (Figure 4A). However, on the basis of fatty acids (Figure 4B), they clustered with both *B. ceti* porpoise type B1/94 and *B. ceti* Atlantic dolphin type B14/94 strains. In spite of this, all *B. ceti* strains remained as a separate cluster from *B. pinnipedialis* seal B2/94 strain and other *brucellae* from terrestrial animals.

**Figure 5:**
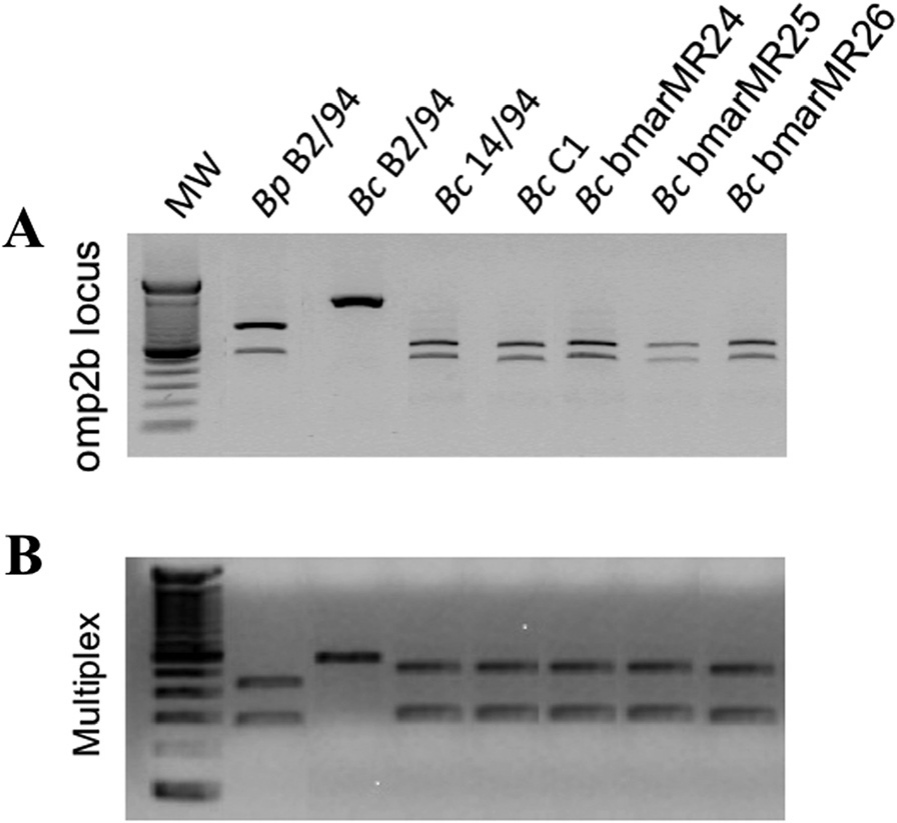
**Molecular characterization of** ***Brucella*** **isolates by PCR-RFLP of** ***omp2b*** **locus and Multiplex PCR.** Panel **A**: PCR-RFLP of *omp2b* locus, showing a common haplotype for the three *B. ceti* Mediterranean isolates (lanes 6–8), shared with that identified in all *B. ceti* Atlantic dolphin strains, irrespective of their geographic origin (lanes 4 and 5), but different from *B. pinnipedialis* and *B. ceti* porpoise reference (lanes 2 and 3, respectively) haplotypes. Panel **B**. Multiplex PCR using two primer pairs, identifying Mediterranean isolates as similar to those of Atlantic dolphin origin. Lane 1, 100 bp DNA ladder (Invitrogen Ltd.); lane 2, *B. pinnipedialis* B2/94 reference seal strain; lane 3, *B. ceti* B1/94 porpoise reference strain; lane 4, *B. ceti* B14/94 reference strain isolated from Atlantic dolphin*;* lane 5, *B. ceti* C1 strain isolated from a dolphin in the Cantabric sea (North Spain); lanes 6, 7 and 8, *B. ceti* Mediterranean isolates (bmarMR24, bmarMR25 and bmarMR26, respectively).

**Figure 6:**
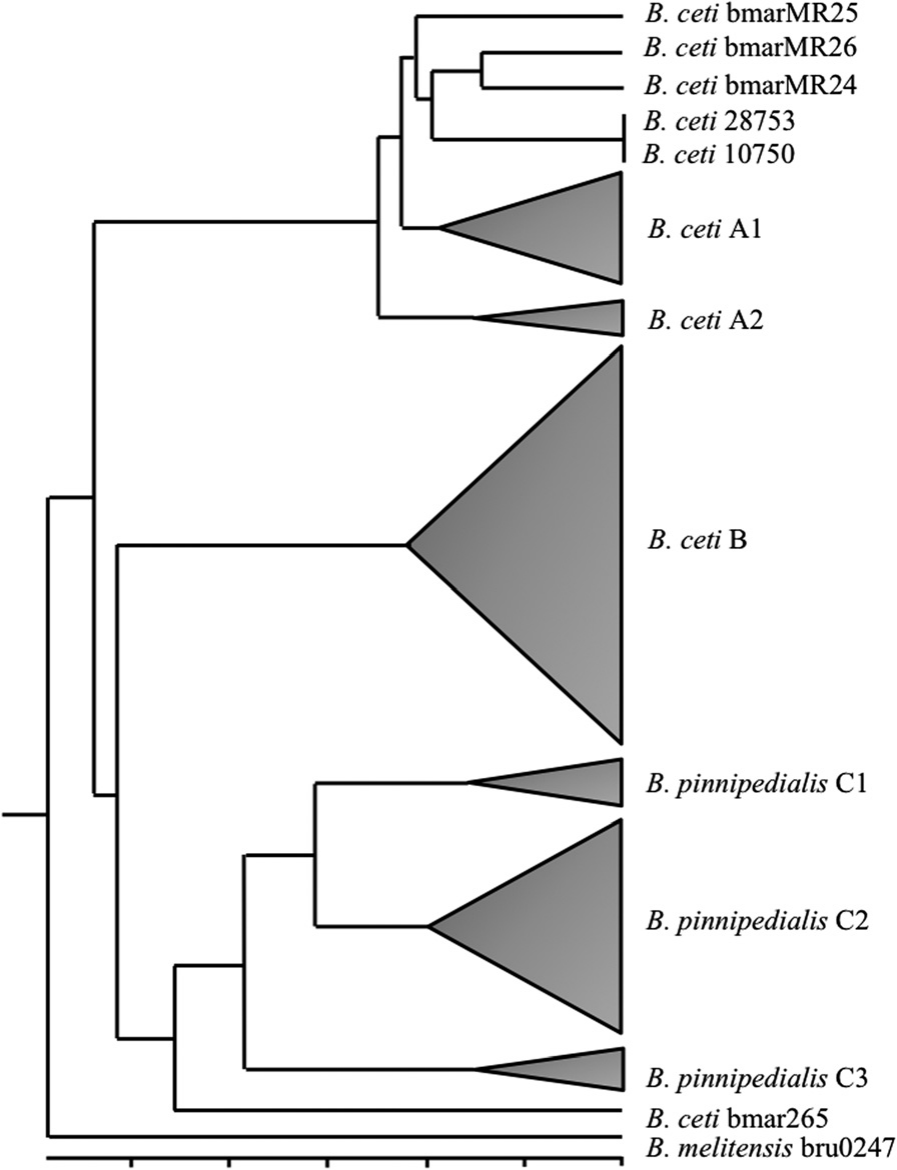
**Cladogram based on MLVA16 analysis of** ***Brucella*** **isolates from marine mammals (** http://mlva.upsud.fr/brucella/ **).** The dispersion of the various *Brucella* strains is depicted as cones proportional to the number of strains analyzed. Notice than the five *B. ceti* Mediterranean strains (three from this work and two reported by Garofolo et al. [15]) cluster together in a single group in a clade close to the Atlantic isolates (A1 and A2), and far from the *B. ceti* porpoise type (B) and *B. pinnipedialis* (C1, C2, C3). *B. ceti* bmar265 (human isolate from New Zealand) does not correspond to the *B. ceti* group and it is ST27. *B. melitensis* was used as an out-group for the analysis.

**Figure 7:**
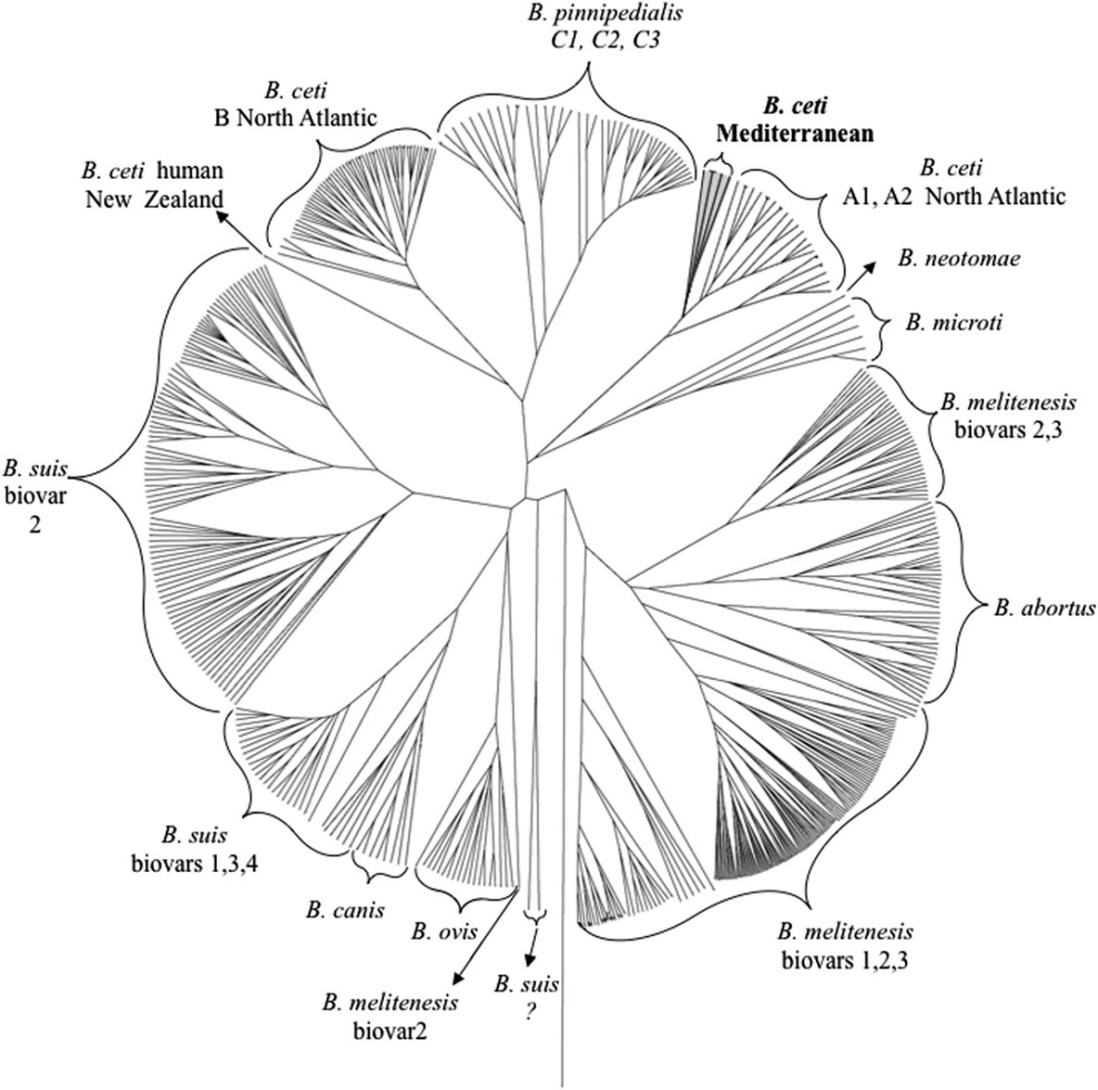
***Brucella*** **phylogenetic tree based on MLVA16 analysis of all the various** ***Brucella*** **species and strains (** http://mlva.upsud.fr/brucella/ **).** Notice that the three Western (Spanish) and the Eastern (Italian) *B. ceti* Mediterranean strains cluster together in a single group (shadowed in gray) in a clade close to the Atlantic isolates (A1 and A2), all belonging to ST 26.

The PCR-RFLP of the *omp2b* locus confirmed also the close relationship of the three Mediterranean isolates of *B. ceti*, resulting in a common haplotype identical in all strains, irrespective of their geographic origin (Figure 5A). Similarly, the multiplex PCR using two primer pairs identified the Mediterranean isolates as similar to the Atlantic dolphin type *B. ceti* B14/94 strain (Figure 5B). The MLVA16 phylogenetic analysis showed that the three *B. ceti* Spanish Mediterranean strains clustered together in a distinct clade together with the two reported *B. ceti* isolated in the Mediterranean Italian littoral [15] and close to the Atlantic A1 dolphin cluster (Figure 6). The distinct topology of the Mediterranean *B. ceti* isolates is maintained even when a comprehensive phylogenetic analysis against the complete *Brucella* MLVA16 base data is performed (Figure 7). As expected, the *B ceti* Mediterranean isolates MLVA 16 patterns were identical to strains previously typed by MLSA as ST 26.

## Discussion

During the last decade *B. ceti* strains have been isolated from stranded dolphins of both the Atlantic and Pacific Oceans [5]. Information about *B.ceti* strains from the Mediterranean Sea is scarce. Recently, a *B. ceti* strain showing phenotypic characteristics of Atlantic *B. ceti* strains was isolated from a striped dolphin in the Tyrrhenian littoral of Italy [11]. Also, two other *B. ceti* strains have been characterized from the Italian Southern Apulian Coast [15]. MLVA analysis of the two isolates assigned them to a novel genotype within cluster A. Here we confirm and extend these observations, endorsing the presence of *B. ceti* infecting and causing pathology in at least two dolphin species (*S. coeruleoalba* and *T. truncatus*) inhabiting the Mediterranean Sea. The multilocus sequence analysis of these three *B. ceti* Mediterranean strains indicates that they also belong to the dolphin type of the ST26 cluster.Up to now, all *B. ceti* Mediterranean strains stem in a separate branch from the main MLVA16 A1 and A2 clusters of *B. ceti* isolates from dolphins inhabiting the Atlantic Ocean. Although the number of analysed Mediterranean *B. ceti* strains by MLVA16 is still low to draw a definitive branching order, the taxonomical position of these newly defined strains [11] is supported by proteome analysis and, to a less extent, by fatty acids analysis, two robust techniques used in bacterial taxonomy [33] and useful for characterization of *Brucella* [9-11,24,31,32]. Therefore, it seems that the *B. ceti* isolates described here belong to a particular genotype prevalent in the Mediterranean Sea. The close relationship between Mediterranean and Atlantic *B. ceti* strains keeps important parallelism with the phylogenetic and taxonomical studies on the striped and bottlenose dolphin populations in both seas. Indeed, there is strong evidence of dispersal of bottlenose dolphin populations between both seas, keeping a population structure and genetic diversity in concordance with the boundaries that coincide with transitions between different habitat regions [34]. In the case of striped dolphins, there is also evidence of some genetic flow between the Mediterranean and Atlantic populations; albeit*,* this is significantly more restricted than in bottlenose dolphins. This is mainly due to ecological and behavioral factors that limit the exchange between these two *S. coeruleoalba* populations across Gibraltar Strait [35]. Considering this, the phenotypic and genetic structure from both North Atlantic and Mediterranean *B. ceti* isolates is not unexpected, as populations of *S. coeruleoalba* and *T. truncatus* share similar habitats and feed resources, and may then share various microorganisms, including *B. ceti*.

Infection by *B. ceti* is common in cetaceans but only a small proportion of infected cetaceans display clinicopathological signs associated to brucellosis, suggesting that many infected cetaceans overcome infection, perhaps remaining as carriers and potential *Brucella* shedders [5]. This is in sharp contrast with the absence of obvious disease in seals or walrus infected with *B. pinnipedialis* [36]. Several clinico-pathological entities have been associated to *B. ceti* infection in a relevant fraction of the stranded dolphins and porpoises [5], with neurobrucellosis being one of the most significant signs present [8,12,11]. One of our Mediterranean striped dolphins (N-301/12) presented unequivocally *B. ceti* induced non-suppurative meningoencephalitis with *B. ceti* being isolated from the CNS, and regarded as the primary cause of death. This is in agreement with the suggested higher susceptibility of this dolphin species for developing neurobrucellosis in comparison to other cetaceans [12].

*B. ceti* has been found to invade joints and cause chronic inflammatory lesions, and has been frequently isolated from these lesions in cetaceans [10,12,37]. In line with these observations, a causal relationship was hypothesized between the discospondylitis of the peduncle observed in the bottlenose dolphin case (N-275/12) and the *B.ceti* strain isolated from that lesion. This discospondylitis could be causing a disabling condition, but this lesion was probably not live-threatening. Primary cause of death in this dolphin was attributed to a mycotic encephalitis caused by *Cunninghamella bertholletiae*.

Similarly, death of the other striped dolphin (N-372/09) was linked to CeMV-related encephalitis and not to *B. ceti* infection (isolated retrospectively only from spleen, without evidence of lesions related to *Brucella*). CeMV infections of the brain have been unambiguously linked with epizootic disease and deaths of Mediterranean striped dolphins in the past [17,38].

As a corollary to these findings, it appears mandatory to establish adequate differential diagnoses for the aetiological agents that may affect disoriented live-stranded dolphins. This is becoming even more relevant when a number of cetaceans in the Mediterranean Sea show a rapid decline [39], mainly due to by-catch, the presence of pollutant contamination, and the pressure of infectious diseases that threaten the health of free-ranging cetaceans [40]. Brucellosis, as a contagious disease, can be an additional factor hampering the conservation efforts of cetaceans at local and global scale. Our study confirms the relevance of *B. ceti* as a cetacean pathogen in the Mediterranean, the severity of pathological signs in stranded *S. coeruleoalba* dolphins, and gives insight on the phylogenetic structure of these *B. ceti* Mediterranean isolates. The Mediterranean *B. ceti* strains isolated so far form a distinct phylogenetic cluster, close to that of *B. ceti* strains isolated from dolphins inhabiting the Atlantic Ocean. In addition to seriously compromising the wellbeing of marine mammals, the *B. ceti* strains possess all the current molecular virulence determinants and therefore, are potential pathogens for other animals, including humans [5].

## Conclusions

*B. ceti* has been isolated for the first time from the Spanish Mediterranean Sea, expanding the known range of this species. Neurobrucellosis with non-suppurative meningoencephalomyelitis in a striped dolphin and spondylitis in a bottlenose dolphin were the main clinicopathological features of these cases. In a third case, *B. ceti* was isolated from the spleen of a striped dolphin. The *omp2b* haplotype was common for all three *B. ceti* Mediterranean isolates and multiplex characterization showed that they were similar to the Atlantic dolphin type *B. ceti* B14/94 strain. Following MLVA16 analysis the three *B. ceti* Spanish Mediterranean strains clustered together in a distinct clade with the two reported *B. ceti* isolated in the Mediterranean Italian littorals, and close to the Atlantic A1 dolphin cluster. This taxonomical position was supported by protein and fatty acid analyses. Collecting appropriate samples for testing for *Brucella* has to be included in necropsy protocols in stranded dolphins.

### Ethics statement

The work did not include experimental procedures. Handling of live and dead cetaceans (species included in CITES 2 list) was done with official governmental permission. Medical treatments on live dolphins were applied following established procedures for these species.

## Additional files

Additional file 1: Table S1. MLVA-16 genetic profiles for the three B. ceti isolates*. Additional file 2: Table S2. Protein molecular weights peaks of the different Brucella strains determined by MALDI-TOF. Additional file 3: Table S3. Fatty acids determined by gas liquid chromatography of different Brucella strains.
